# Surface roughness of enamel and cention N after toothpaste brushing: An *ex vivo* study

**DOI:** 10.6026/973206300221660

**Published:** 2026-03-31

**Authors:** Seema Dixit, Vidya Chawla, Pulkit Gupta, Anshdeep Singh, Priya Pundir, Arpit Arora

**Affiliations:** 1 Department of Conservative Dentistry and Endodontics, Seema Dental College and Hospital, Rishikesh, Uttarakhand, India

**Keywords:** Abrasivity, enamel, powered brushing, restorative materials, surface roughness

## Abstract

Prolonged exposure to powered brushing, especially with high-abrasive toothpastes, can significantly increase surface roughness, affecting both enamel and restorative materials. Therefore, it is of interest to assess the cumulative abrasive effects of different toothpaste formulations under extended powered brushing conditions. Thus, we show the need for a balanced approach to plaque removal and surface preservation, highlighting the importance of selecting appropriate toothpaste formulations to minimize adverse effects on oral and restorative surfaces.

## Background:

Tooth brushing with dentifrice is the most widely recommended method for daily plaque control and oral hygiene maintenance. However, the mechanical and abrasive action associated with brushing can gradually alter the surface characteristics of enamel and restorative materials, which may lead to undesirable effects [1]. Increased surface roughness caused by brushing facilitates plaque retention, staining and biofilm maturation, which may ultimately compromise esthetics, periodontal health and the longevity of dental restorations. These changes in surface properties are of significant concern in preventive and restorative dentistry [2]. As such, understanding the interaction between different toothpastes and both natural tooth structure and restorative materials has become an important aspect of maintaining dental health and the longevity of restorative treatments [3]. Tooth wear is a multifactorial condition that arises from the combined effects of mechanical abrasion, chemical erosion and tribological interactions at the tooth surface. The abrasive particles suspended in the toothpaste slurry play a key role during tooth brushing, as they act between the bristles and the tooth or restorative surface [4]. Various factors influence the wear response, including abrasive type and concentration, particle morphology, brushing force, time and bristle stiffness. The wear susceptibility of enamel and dentine differs, with dentine being more vulnerable to wear, particularly at exposed root surfaces. Studies have shown that the concentration of toothpaste can affect wear patterns, with higher concentrations leading to marked increases in dentine wear, while enamel wear remains relatively stable [5]. This underscores the substrate- dependent nature of toothpaste abrasivity and its varied effects on different tooth surfaces. Quantifying the abrasivity of toothpaste is typically done using relative dentin abrasivity (RDA) values, with some studies also considering relative enamel abrasivity (REA). International standards define acceptable RDA ranges and categorize abrasivity into low, medium, or high levels [6]. However, products within these limits may still produce distinct patterns of tissue loss and surface roughening.

Profilometric methods, which assess surface roughness parameters such as Ra (arithmetic mean roughness), provide more qualitative and quantitative information about the topographical changes caused by tooth brushing. These methods, when combined with RDA measurements, offer a more complete picture of the clinical behaviour of different dentifrices [7]. Recent research has focused on specific categories of toothpaste, such as herbal, whitening, charcoal-containing and conventional fluoridated formulations. Herbal toothpastes often incorporate plant-derived extracts and may use alternative abrasive systems. Some studies have shown that certain herbal formulations can increase surface roughness and alter the mechanical properties of aesthetic restorative materials [8]. Whitening toothpastes, which typically rely on higher abrasive loads and additional chemical agents to remove extrinsic stains, are often found to exhibit medium to high RDA values. Although these products remain below the upper safety threshold recommended by professional bodies, they may cause significant surface texture changes on enamel or restorative materials depending on their abrasive system [9]. Charcoal-based toothpastes have gained popularity due to their perceived natural whitening effect. However, multiple in vitro and clinical studies have associated charcoal-containing toothpastes with increased enamel roughness and abrasion compared to conventional fluoridated dentifrices [10]. Profilometric and microscopic analyses consistently demonstrate that activated charcoal toothpastes tend to produce more pronounced roughness and surface loss, raising concerns about their long-term impact on enamel integrity. Conventional fluoridated toothpastes remain the cornerstone of caries prevention, offering effective cleaning with abrasivity levels considered safe for long-term use. These formulations also provide fluoride delivery, which contributes to remineralization and caries control while balancing stain removal against the risk of excessive tissue loss [11]. Restorative materials, like Cention N, are exposed daily to tooth brushing abrasion and may undergo surface changes that affect their clinical performance. Increased roughness on restorative surfaces can enhance biofilm accumulation, stain uptake and wear, potentially leading to marginal degradation and a reduced lifespan of the restoration [12]. Cention N is an "alkasite" bulk-fill restorative material designed as a tooth- colored alternative for stress-bearing posterior restorations. Its formulation includes a resin matrix combined with alkaline glass fillers, capable of releasing calcium, fluoride and hydroxide ions, providing both ion release and acid-neutralizing properties. Due to its specific filler technology, Cention N may interact differently with the abrasive components of toothpastes when compared to natural enamel [13]. Therefore, it is of interest to determine the impact of different toothpaste categories on the surface roughness of enamel and Cention N and to identify which category leads to the least adverse surface alterations, providing valuable insights for clinicians and patients on the best choices for maintaining both oral and restorative health.

## Methodology:

The present *ex vivo* study was conducted in the Department of Conservative Dentistry and Endodontics at Seema Dental College and Hospital, Rishikesh, Uttarakhand, in collaboration with The Indian Institute of Technology, Roorkee, Uttarakhand. The aim of the study was to evaluate and compare the surface roughness of enamel and Cention N after brushing with various categories of toothpastes, using a profilometer. Sample size estimation was performed using G Power software (version 3.0) for a One-way ANOVA test. The required sample size was calculated for an alpha of 0.05, a power of 80% and an effect size of 0.45, which was derived from a similar study on surface roughness. The estimated total sample size was 60, divided into four groups of 15 samples each. This sample size was determined based on the F-tests for ANOVA (Fixed effects, omnibus, one-way). The analysis showed a critical F value of 2.7694, with a numerator df of 3 and a denominator df of 56. The actual power achieved was 0.8165, which was sufficient for the study's purposes. A total of 60 freshly extracted intact and caries-free human permanent teeth were collected from patients undergoing extraction for orthodontic or periodontal reasons. The inclusion criteria for the selection of teeth were as follows: intact teeth with no pre-existing caries, restorations or developmental anomalies; complete root formation; and freshly extracted teeth (within 24 hours of extraction). Immediately after extraction, extraneous soft tissues, superficial debris and calculus were removed from the root surfaces using an ultrasonic scalar. Adhering to the CDC Guidelines for infection control in dental health-care settings (2003) for sample preparation, all teeth were disinfected with 5.25% sodium hypochlorite (NaOCl) solution for 10 minutes, followed by thorough rinsing with distilled water. To ensure proper sterilization, the teeth were autoclaved at 121°C for 15 minutes at 15 psi pressure. The materials, armamentarium and equipment used in the study included the following. For sample preparation, freshly extracted human permanent teeth were used. An ultrasonic scaler, high-speed airotor, #245 bur, straight fissure bur and self-polymerizing acrylic resin were employed for cleaning and mounting the teeth. The teeth were set in a pre- fabricated metal mold and Vaseline was applied to prevent any unwanted adhesion. For restorative material, Cention N, a tooth-colored bulk- fill restorative material, was utilized. The toothpastes used in the study included herbal toothpaste, teeth whitening toothpaste, charcoal toothpaste and conventional fluoridated toothpaste. Brushing was performed using an electric-powered toothbrush with a soft-bristled brush head. For the incubation process, an incubator was used and surface roughness was measured using an optical surface profilometer. These tools and materials were essential for conducting the study and ensuring accurate and reliable results regarding the effects of different toothpastes on the surface roughness of enamel and Cention N. Following sterilization, Class V cavities were prepared on the buccal surface of each of the 60 teeth at the cemento-enamel junction (CEJ) using a #245 bur. Each cavity was restored with Cention N according to the manufacturer's instructions. Once the restorations were placed, they were finished and polished using finishing burs to ensure a smooth surface. To standardize the tooth samples, the teeth were decoronated 2 mm below the CEJ using a straight fissure bur under water spray, achieving a flush restoration that matched the tooth surface. The polymer and monomer of cold cure resin were mixed in a silicon bowl with the help of an agate spatula. Each tooth was then embedded in self-polymerizing acrylic resin using a pre-fabricated metal mold. The buccal and labial surfaces of the tooth, containing both enamel and restoration, were positioned facing upward and left free from resin to allow direct exposure to brushing. The embedded portion of the tooth in resin served as a holder during mechanical brushing and surface roughness assessment. After the polymerization process, the samples were cleaned with distilled water and air-dried, ensuring that they were ready for the next stage of the study.

The 60 samples were divided randomly into four groups of 15 samples each based on the toothpaste category:

[1] Group I: Herbal Toothpaste

[2] Group II: Teeth Whitening Toothpaste

[3] Group III: Charcoal-Containing Toothpaste

[4] Group IV: Conventional Fluoridated Toothpaste

All samples in the four groups underwent mechanical brushing using a standardized electric toothbrush under controlled conditions to ensure consistency:

[1] Brushing frequency: Twice daily (morning and evening)

[2] Brushing duration: 2 minutes per brushing session

[3] Total duration: 3 months (approximately 360 brushing cycles)

[4] Toothbrush type: Soft-bristled powered toothbrush

Before each brushing session, a fresh pea-sized amount of the designated toothpaste was applied to the bristles for each sample.

Between brushing sessions, all samples were stored in sterile saline in an incubator at a temperature of 37°C. This environment was designed to simulate physiological conditions and prevent dehydration of the tooth samples. Surface roughness was assessed for all specimens using a surface profilometer. After completing the 3-month brushing protocol (2 minutes following the final brushing session), surface roughness measurements were recorded using a surface profilometer. The profilometer was calibrated according to the manufacturer's specifications before each measurement session to ensure accuracy and consistency.

For each sample, surface roughness measurements were taken at two distinct sites:

[1] Enamel surface: Measurements were taken on the exposed coronal enamel, away from the restoration margins, to assess the effect of brushing on natural tooth enamel.

[2] Cention N surface: Measurements were taken on the surface of the restoration exposed to brushing to evaluate how brushing impacted the restorative material.

The profilometer was properly oriented on the required sites to obtain the necessary readings, ensuring that both the enamel and Cention N surfaces were accurately measured for roughness.

## Results:

Ra (arithmetic mean roughness) and Rq (root mean square roughness) are primary amplitude parameters measured by profilometers to quantify surface texture in dental material studies. Ra represents the arithmetic average of absolute deviations from the mean line along the profilometer's sampling length, providing a general indicator of surface smoothness insensitive to isolated peaks or valleys. Rq, calculated as the square root of the mean of squared deviations, emphasizes taller peaks and deeper valleys due to squaring, making it more sensitive to extreme irregularities. Table 1 (see PDF) shows the association between toothpaste groups and surface roughness parameters, with significant differences observed for both Ra (µm) and Rq (µm). The results indicate that different toothpaste formulations lead to varying surface roughness values across the groups, as evidenced by the statistical analysis. Specifically, the Ra values for Charcoal, Conventional Fluoridated, Herbal and Teeth Whitening toothpaste groups were significantly different (p<0.05), with the Herbal group exhibiting the highest roughness. Similarly, the Rq values also differed significantly (p<0.05) among the toothpaste groups, with Teeth Whitening toothpaste showing the highest Rq values. These findings suggest that toothpaste selection can have a substantial impact on the surface roughness of both enamel and restorative materials. Table 2 (see PDF) shows significant differences (p<0.05) between the toothpaste groups in both Ra (µm) and Rq (µm) values for enamel, with the Herbal toothpaste group exhibiting the highest surface roughness values, followed by Teeth Whitening, Charcoal and Conventional Fluoridated toothpaste. The Kruskal-Wallis test revealed significant differences in the Ra (µm) values across the four toothpaste groups (χ^2^ = 32.248, p < 0.001). Pairwise comparisons indicated that Charcoal, Conventional Fluoridated and Herbal toothpaste groups exhibited significant differences in surface roughness, with Conventional Fluoridated toothpaste showing the lowest surface roughness. However, no significant difference was found between Charcoal and Herbal toothpaste, as well as between Charcoal and Teeth Whitening toothpaste Table 3 (see PDF). Pairwise Comparison of Surface Roughness of Enamel and Cention N in Different Toothpaste are shown in Table 4 (see PDF).

The variable Ra (µm) was not normally distributed in the 4 subgroups of the variable Toothpaste Group. Thus, non-parametric tests (Kruskal Wallis Test) were used to make group comparisons. The mean (SD) of Ra (µm) in the Toothpaste Group: Charcoal group was 4.23 (0.45). The mean (SD) of Ra (µm) in the Toothpaste Group: Conventional fluoridated group was 3.52 (0.49). The mean (SD) of Ra (µm) in the Toothpaste Group: Herbal group was 4.32 (0.67). The mean (SD) of Ra (µm) in the Toothpaste Group: Teeth Whitening group was 4.27 (0.38). The median (IQR) of Ra (µm) in the Toothpaste Group: Charcoal group was 4.3 (3.86-4.5). The median (IQR) of Ra (µm) in the Toothpaste Group: Conventional fluoridated group was 3.37 (3.15-3.82). The median (IQR) of Ra (µm) in the Toothpaste Group: Herbal group was 4.37 (3.97-4.66). The median (IQR) of Ra (µm) in the Toothpaste Group: Teeth Whitening group was 4.3 (4.03-4.52). The Ra (µm) in the Toothpaste Group: Charcoal ranged from 3.4 - 5.01. The Ra (µm) in the Toothpaste Group: Conventional fluoridated ranged from 2.7-4.42. The Ra (µm) in the Toothpaste Group: Herbal ranged from 3.04 - 5.65. The Ra (µm) in the Toothpaste Group: Teeth Whitening ranged from 3.45 - 5.17. There was a significant difference between the 4 groups in terms of Ra (µm) (χ^2^ = 32.248, p = <0.001), with the median Ra (µm) being highest in the Toothpaste Group: Herbal group (Table 5 - see PDF). Further complements this data by showing the distribution density of Ra (µm) values across the toothpaste groups for the full sample. The density plot provides a more detailed view of the distribution, allowing for a better understanding of how the surface roughness values are spread within each group. Together, Figure 1 (see PDF) offers a comprehensive view of the impact of different toothpaste formulations on the surface roughness of the enamel and restorative materials. The Kruskal-Wallis test revealed significant differences in the Ra (µm) values across the four toothpaste groups for Cention N (χ^2^ = 10.488, p = 0.015). Pairwise comparisons indicated that Charcoal toothpaste and Conventional Fluoridated toothpaste exhibited significantly different surface roughness, with Charcoal showing higher roughness compared to Conventional Fluoridated toothpaste (adjusted p = 0.024). However, no significant difference was found between Charcoal and Herbal, or Charcoal and Teeth Whitening. Interestingly, Conventional Fluoridated and Herbal toothpastes also showed no significant difference in surface roughness (adjusted p = 0.150). Teeth Whitening toothpaste showed a significant difference from Conventional Fluoridated tooth paste (adjusted p = 0.046), but no significant difference was observed when compared with Herbal toothpaste (adjusted p = 0.998). These results highlight the distinct effects of different toothpaste formulations on the surface integrity of Cention N, with Charcoal and Teeth Whitening formulations causing more significant surface roughness compared to the Conventional Fluoridated toothpaste. Table 6 (see PDF) shows association between Toothpaste Group' and' Ra (µm)' in Substrate: Cention N. Table 7 (see PDF) shows comparison of Surface Roughness (Ra) of Cention N Treated with Different Toothpastes. Figure 2 (see PDF) shows association Between Toothpaste Group and Ra (µm) Full sample Distribution Density.

The variable Ra (µm) was not normally distributed in the 4 subgroups of the variable Toothpaste Group. Thus, non-parametric tests (Kruskal Wallis Test) were used to make group comparisons. The mean (SD) of Ra (µm) in the Toothpaste Group: Charcoal group was 4.17 (0.46). The mean (SD) of Ra (µm) in the Toothpaste Group: Conventional fluoridated group was 3.59 (0.49). The mean (SD) of Ra (µm) in the Toothpaste Group: Herbal group was 4.00 (0.61). The mean (SD) of Ra (µm) in the Toothpaste Group: Teeth Whitening group was 4.12 (0.34). The median (IQR) of Ra (µm) in the Toothpaste Group: Charcoal group was 4.15 (3.81-4.53). The median (IQR) of Ra (µm) in the Toothpaste. Group: Conventional fluoridated group was 3.48 (3.18-3.91). The median (IQR) of Ra (µm) in the Toothpaste Group: Herbal group was 4.29 (3.52-4.43). The median (IQR) of Ra (µm) in the Toothpaste Group: Teeth Whitening group was 4.19 (3.89-4.35). The Ra (µm) in the Toothpaste Group: Charcoal ranged from 3.4 - 5. The Ra (µm) in the Toothpaste Group: Conventional fluoridated ranged from 3.06 - 4.42. The Ra (µm) in the Toothpaste Group: Herbal ranged from 3.04-4.87.The Ra (µm) in the Toothpaste Group: Teeth Whitening ranged from 3.45 - 4.6. There was a significant difference between the 4 groups in terms of Ra (µm) (χ^2^ = 10.488, p=0.015), with the median Ra (µm) being highest in the Toothpaste Group: Herbal group. Table 8 (see PDF) shows surface Ra values of Cention N while Table 9 (see PDF) shows Ra (µm) values for each toothpaste group. Pairwise comparison of subcategories of toothpaste group has been shown in Table 10 (see PDF).

The variable Ra (µm) was normally distributed in the 4 subgroups of the variable Toothpaste Group. Thus, parametric tests (One-Way ANOVA) were used to make group comparisons. The mean (SD) of Ra (µm) in the Toothpaste Group: Charcoal group was 4.28 (0.45). The mean (SD) of Ra (µm) in the Toothpaste Group: Conventional fluoridated group was 3.44 (0.50). The mean (SD) of Ra (µm) in the Toothpaste Group: Herbal group was 4.64 (0.57).The mean (SD) of Ra (µm) in the Toothpaste Group: Teeth Whitening group was 4.41 (0.38). The median (IQR) of Ra (µm) in the Toothpaste Group: Charcoal group was 4.36 (4.11-4.44). The median (IQR) of Ra (µm) in the Toothpaste Group: Conventional fluoridated group was 3.25 (3.11-3.72). The median (IQR) of Ra (µm) in the Toothpaste Group: Herbal group was 4.59 (4.2-5.01). The median (IQR) of Ra (µm) in the Tooth paste Group: Teeth Whitening group was 4.37 (4.07-4.71). The Ra (µm) in the Tooth paste Group: Charcoal ranged from 3.45 - 5.01. The Ra (µm) in the Toothpaste Group: Conventional fluoridated ranged from 2.7 - 4.41. The Ra (µm) in the Toothpaste Group: Herbal ranged from 3.81 - 5.65. The Ra (µm) in the Toothpaste Group: Teeth Whitening ranged from 3.98 - 5.17. There was a significant difference between the 4 groups in terms of Ra (µm) (F = 17.583, p=<0.001), with the mean Ra (µm) being highest in the Toothpaste Group: Herbal group. Table 11 (see PDF) shows association Between Toothpaste Group and Rq (µm) Cention N Surface. Table 12 (see PDF) shows the association Between Toothpaste Group and Rq (µm) Cention N Surface Distribution Density. The variable Rq (µm) was normally distributed in the 4 subgroups of the variable Toothpaste Group. Thus, parametric tests (One-Way ANOVA) were used to make group comparisons. Table 13 (see PDF) & Table 14 (see PDF) summarizes the mean (SD) of Rq (µm) of the 4 groups in the Toothpaste Group. The mean (SD) of Rq (µm) of in the Toothpaste Group: Charcoal group was 4.99 (0.47). The mean (SD) of Rq (µm) in the Toothpaste Group: Conventional fluoridated group was 4.34 (0.55). The mean (SD) of Rq (µm) in the Toothpaste Group: Herbal group was 4.73 (0.64). The mean (SD) of Rq (µm) in the Toothpaste Group: Teeth Whitening group was 4.94 (0.48). The median (IQR) of Rq (µm) in the Toothpaste Group: Charcoal group was 4.81(4.61-5.25). The median (IQR) of Rq (µm) in the Toothpaste Group: Conventional fluoridated group was 4.26 (3.96-4.69). The median (IQR) of Rq (µm) in the Toothpaste Group: Herbal group was 4.78 (4.28-5.2). The median (IQR) of Rq (µm) in the Toothpaste Group: Teeth Whitening group was 4.9 (4.62-5.15). The Rq (µm) in the Toothpaste Group: Charcoal ranged from 4.45 - 5.98. The Rq (µm) in the Toothpaste Group: Conventional fluoridated ranged from 3.47 - 5.33. The Rq (µm) in the Toothpaste Group: Herbal ranged from 3.73 - 5.88. The Rq (µm) in the Toothpaste Group: Teeth Whitening ranged from 4.13 - 6.09. There was a significant difference between the 4 groups in terms of Rq (µm) (F=4.423, p = 0.007), with the mean Rq (µm) being highest in the Toothpaste Group: Charcoal group.

Post-Hoc pairwise tests for ANOVA performed using Tukey HSD method. Table 11 (see PDF) & 12 (see PDF) summarizes the Rq (µm) values for Cention N across the four toothpaste groups. Significant differences were observed between Conventional Fluoridated and Charcoal and between Herbal and Conventional Fluoridated. Teeth Whitening showed no significant difference from Charcoal but had notable differences from other groups. The variable Rq (µm) was normally distributed in the 4 subgroups of the variable Toothpaste Group. Thus, parametric tests (One-Way ANOVA) were used to make group comparisons (Table 10 - see PDF). The mean (SD) of Rq (µm) in the Toothpaste Group: Charcoal group was 5.19 (0.63). The mean (SD) of Rq (µm) in the Toothpaste Group: Conventional fluoridated group was 4.45 (0.48). The mean (SD) of Rq (µm) in the Toothpaste Group: Herbal group was 5.16 (0.31). The mean (SD) of Rq (µm) in the Toothpaste Group: Teeth Whitening group was 5.56 (0.52). The median (IQR) of Rq (µm) in the Toothpaste Group: Charcoal group was 5.18 (4.94-5.67). The median (IQR) of Rq (µm) in the Toothpaste Group: Conventional fluoridated group was 4.61(4.24-4.84). The median (IQR) of Rq (µm) in the Toothpaste Group: Herbal group was 5.21 (4.96-5.38). The median (IQR) of Rq (µm) in the Tooth paste Group: Teeth Whitening group was 5.54 (5.11-5.89). The Rq (µm) in the Toothpaste Group: Charcoal ranged from 3.79 - 5.94. The Rq (µm) in the Toothpaste Group: Conventional fluoridated ranged from 3.62-5.19. The Rq (µm) in the Toothpaste Group: Herbal ranged from 4.67 - 5.65. The Rq (µm) in the Toothpaste Group: Teeth Whitening ranged from 4.93 - 6.39. There was a significant difference between the 4 groups in terms of Rq (µm) (F = 13.175, p=<0.001), with the mean Rq (µm) being highest in the Toothpaste Group: Teeth Whitening group.

## Ra (µm):

[1] Highest: Herbal = 4.32±0.67

[2] Lowest: Conventional fluoridated = 3.52±0.49

[3] Significant differences (χ^2^=32.248, p<0.001)

## Rq (µm):

[1] Highest: Teeth Whitening=5.25±0.59

[2] Lowest: Conventional fluoridated = 4.39±0.51

[3] Significant differences (F=13.706, p< 0.001)

## Discussion:

Surface roughness plays a pivotal role in the long-term success of restorative materials and natural tooth structure. Roughened surfaces are more prone to plaque retention, which can lead to gingival inflammation, staining and secondary caries over time. Previous studies have demonstrated that surface roughness significantly influences plaque accumulation, staining susceptibility, wear progression and overall restoration longevity. The current study reinforces the importance of maintaining smooth surfaces, as roughened enamel and restorative materials are more likely to foster plaque build-up, which in turn may negatively affect both aesthetic and biological outcomes in restorative dentistry. The findings of this study suggest that routine oral hygiene practices, including the use of specific toothpaste formulations, should be considered as integral components of restorative success, rather than isolated patient factors. The vulnerability of enamel to mechanical abrasion is closely tied to its microstructural organization. Although enamel is highly mineralized and exhibits high hardness, its prismatic structure contains interfaces that are susceptible to repetitive mechanical stress, such as brushing. In this study, enamel consistently demonstrated higher surface roughness values (Ra and Rq) along with Cention N across all toothpaste groups, confirming its greater susceptibility to abrasion. This finding is consistent with the study by Enax *et al.* (2023) [14], who also highlighted enamel's susceptibility to abrasion. Although, Kaptan *et al.* (2023) [15], highlighted about Cention N being resistant to mechanical wear, it did show surface roughness post brushing. Powered toothbrushes are commonly recommended due to their superior plaque removal efficiency. However, their interaction with restorative materials warrants careful consideration. The present study simulated extended powered brushing conditions to assess cumulative surface alterations, revealing that prolonged exposure to powered brushing can intensify abrasive effects, especially when combined with high-abrasive dentifrices. This is consistent with the findings of Jain (2013) [16], who observed higher roughness values with powered toothbrush use compared to manual brushing. The study emphasizes the need to balance effective plaque removal with surface preservation of restorative materials, highlighting the importance of choosing appropriate toothpaste formulations to minimize abrasive effects. Conventional fluoridated toothpaste produced the lowest surface roughness values in both enamel and Cention N, consistent with previous studies that have demonstrated its controlled abrasivity. Fluoride's role in remineralization further enhances enamel's resistance to abrasion, making it the gold standard for oral hygiene, particularly in patients with restorations. Studies such as those by Ganss (2011) [17] support these findings, demonstrating that conventional fluoridated toothpaste offers optimal protection against both wear and surface degradation. This finding is clinically relevant, as it reinforces the recommendation of fluoridated toothpaste for most patients, including those with restorative materials. Herbal toothpaste formulations resulted in the greatest increase in enamel surface roughness. Unlike standardized abrasives, herbal toothpastes contain plant-derived ingredients with variable particle sizes and irregular shapes. This leads to non-uniform abrasion patterns, causing localized surface irregularities. This is in line with Kaur (2025) [18], who found significant roughness increases in both enamel and Cention N surfaces when herbal toothpaste was used. The variability in particle morphology contributes to unpredictable abrasivity, making herbal toothpastes less reliable for patients with enamel wear or extensive restorations. Given the variability in herbal toothpaste formulations, it is crucial for clinicians to tailor recommendations based on individual patient risk profiles. Whitening toothpastes are designed to remove extrinsic stains but may lead to deeper surface defects, particularly in enamel. The present study observed greater increases in Rq values compared to Ra, indicating the formation of deeper, localized surface irregularities. This aligns with Jamwal (2022) [19], who found that whitening toothpastes caused an increase in Ra values, particularly in composite materials. The development of deeper surface irregularities may not be immediately visible but can increase plaque retention over time. Given their potential for deep abrasions, whitening toothpastes should be used cautiously, particularly for long-term applications. This study observed significant difference in the surface roughness of enamel and Cention N amongst all the toothpaste groups corroborating with Ambeth *et al.* (2024) [20]. This study's findings have important clinical implications for restorative dentistry. The pronounced difference in surface roughness between enamel and Cention N emphasizes the need for personalized toothpaste recommendations. Patients with aggressive brushing techniques or extensive restorations should avoid abrasive toothpastes such as herbal or whitening formulations. For patients with Cention N restorations, conventional fluoridated toothpaste is recommended, especially in high-caries-risk patients or those with visible enamel wear. Moreover, clinicians should provide individualized oral hygiene advice and encourage patients to monitor brushing techniques to minimize iatrogenic surface damage and enhance restorative longevity.

## Conclusion:

In conclusion, surface roughness plays a critical role in the long-term success of both natural tooth structure and restorative materials. The findings of this study highlight the importance of selecting appropriate oral hygiene products and brushing techniques to preserve surface integrity and improve clinical outcomes for patients with restorative materials.
